# Association of hospital volume and long-term survival after esophagectomy: A systematic review and meta-analysis

**DOI:** 10.3389/fsurg.2023.1161938

**Published:** 2023-04-21

**Authors:** Qing Wang, Shinji Mine, Motomi Nasu, Tetsu Fukunaga, Shuko Nojiri, Chun-Dong Zhang

**Affiliations:** ^1^ Department of Esophageal and Gastroenterological Surgery, Juntendo University Graduate School of Medicine, Tokyo, Japan; ^2^ Department of Thoracic Surgery, The Fourth Affiliated Hospital of China Medical University, Shenyang, China; ^3^ Medical Technology Innovation Center, Juntendo University, Tokyo, Japan; ^4^ Department of Gastrointestinal Surgery, Graduate School of Medicine, The University of Tokyo, Tokyo, Japan

**Keywords:** esophageal carcinoma, esophagectomy, hospital volume, overall survival, centralization

## Abstract

**Background:**

It remains controversial whether esophageal cancer patients may benefit from esophagectomy in specialized high-volume hospitals. Here, the effect of hospital volume on overall survival (OS) of esophageal cancer patients post esophagectomy was assessed.

**Methods:**

PubMed, Embase, and Cochrane Library were systematically searched for relevant published articles between January 1990 and May 2022. The primary outcome was OS after esophagectomy in high- vs. low-volume hospitals. Random effect models were applied for all meta-analyses. Subgroup analysis were performed based on volume grouping, sample size, study country, year of publication, follow-up or study quality. Sensitivity analyses were conducted using the leave-one-out method. The Newcastle-Ottawa Scale was used to assess the study quality. This study followed the Preferred Reporting Items for Systematic Reviews and Meta-analysis guidance, and was registered (identifier: INPLASY202270023).

**Results:**

A total of twenty-four studies with 113,014 patients were finally included in the meta-analysis. A significant improvement in OS after esophagectomy was observed in high-volume hospitals as compared to that in their low-volume counterparts (HR: 0.77; 95% CI: 0.71–0.84, *P* < 0.01). Next, we conducted subgroup analysis based on volume grouping category, consistent results were found that high-volume hospitals significantly improved OS after esophagectomy than their low-volume counterparts. Subgroup analysis and sensitivity analyses further confirmed that all the results were robust.

**Conclusions:**

Esophageal cancer should be centralized in high-volume hospitals.

## 1. Introduction

Centralization of demanding cancer surgeries to improve the safety and effectiveness of cancer treatment is a topic of ongoing concern in many countries around the world (1–4). Esophagectomy is one of the most complex surgery with high morbidity and mortality, and whether it should be centralized in high-volume hospitals remains controversial (5–9).

Clinical long-term outcomes of esophageal cancer after surgery are usually affected by standardization of surgical procedures, chemotherapy, radiation therapy, molecular targeted therapy and immunotherapy (10–12); moreover, hospital volume also influences mortality after esophagectomy (13). Some previous studies have been reported that esophagectomy for cancer centralized in high-volume hospitals benefited long-term prognosis outcomes (6, 7, 14, 15), whereas, there are also some reports showing inconsistent results (5, 8, 9, 16). Therefore, whether a better long-term overall survival after esophagectomy showing high-volume hospitals remains to be established.

In the present study, we evaluated the influence of high- vs. low-volume hospitals on the long-term OS of patients with esophageal cancer after esophagectomy.

## 2. Materials and methods

### 2.1. Literature search strategy

This systematic review was registered in https://doi.org/10.37766/inplasy2022.7.0023 (identifier: INPLASY202270023) (17). We conducted a systematic search for all relevant articles on the relationship between hospital volume of esophagectomies and long-term OS (17). The search was performed in PubMed, Embase, and Cochrane Library. For example, we combined Medical Subject Headings (MeSH) terms and text terms for the search in PubMed. The following search terms were used: (“esophagectomy” OR “esophageal surgery “ OR “esophageal cancer surgery” OR “esophageal resection” OR “esophageal cancer resection”) AND (“hospital volume” OR “high volume” OR “low volume” OR “healthcare institution size” OR “surgical volume”). We also searched the references of the included studies to search for potentially eligible articles. The last search was completed on May 30, 2022. This study followed the Preferred Reporting Items for Systematic Reviews and Meta-analysis guidance (PRISMA) (17, 18).

### 2.2. Study selection and eligibility criteria

As we previously described, after the retrieval of the relevant articles, they were screened to remove the duplicates (17). All studies were published in English. Search results were screened by two authors (Q.W. and C.D.Z.) independently according to the titles and abstracts. To better reflect modern surgical practices and perioperative management, this study focuses only on articles published after 2002. Next, the retained studies were searched for their full text and further were screened according to the following eligibility criteria: publication in English language; surgery for esophageal carcinoma as the theme; primary outcomes included hospital volume and long-term OS; comparison of OS between high- and low-volume hospitals; original articles with informative data; articles reporting adjusted hazard ratios (HRs) in multivariate analysis; publication before 2002; and articles in which procedural volume was an exact cutoff. Any disagreements were resolved through consultation with the third author (17).

### 2.3. Data extraction

Two authors (QW and CDZ) independently extracted data from the included studies and collated the following information: author, published year, country, study period, population, the unit of exposure (hospital volume), volume classification for hospitals, volume grouping (dichotomies, tertiles, quartiles, quintiles or others) and the longest follow-up and clinical outcomes (OS) (17). Any disagreements were resolved by discussion with the third author. We further assessed the extent of risk adjustment (17).

### 2.4. Study quality evaluation

All included studies were rigorously assessed for methodological quality and risk of bias by two authors (QW and CDZ) by using the Newcastle-Ottawa Scale (17, 19). This scale assesses the quality of studies from three aspects: selection of study population (0–4 points), comparability between groups (0–2 points), and outcome measurement (0–3 points) (17). The total score is 9 points.

### 2.5. Data integration

High-volume hospitals or low-volume hospitals were defined by the authors of the included studies. We used hazard ratios (HRs) in low-volume groups as the reference. If an included study reported more than two surgical volume groups, only the lowest and highest volume groups were compared in the analysis. The primary outcome was OS at the last follow-up, excluding 30-day mortality, 90-day mortality, in-hospital mortality, and postoperative mortality (17).

### 2.6. Statistical analyses

The results were calculated by HRs with 95% confidence intervals (CIs) for long-term outcomes. Heterogeneity among the studies was quantified by the *I*^2^ test, and studies with a statistic of 25%–50% of *I*^2^ were regarded as low heterogeneous, 51%–75% as moderate, and more than 75% as highly heterogeneous (20). Regarding the clinical heterogeneity (inconsistency in pathological staging, therapeutic regimens, and other confounding factors among the studies), we applied random-effect models for all the analyses. To obtain adequate statistical power, subgroup analysis was conducted based on volume grouping category. Then meta-analyses of at least five included studies were performed for different cutoff values (high-volume hospital vs. low-volume hospital). In addition, subgroup analyses in relation to volume group, sample size, study country, year of publication, follow-up or study quality and sensitivity analyses of a leave-one-out method were conducted to verify the results. Funnel plots were used to evaluate potential publication bias. *P* < 0.05 was considered to be statistically significant. All statistical analyses were performed by Review Manager 5.4.1 and Stata 13.1.

## 3. Results

### 3.1. Study selection and characteristics

**Figure 1:**
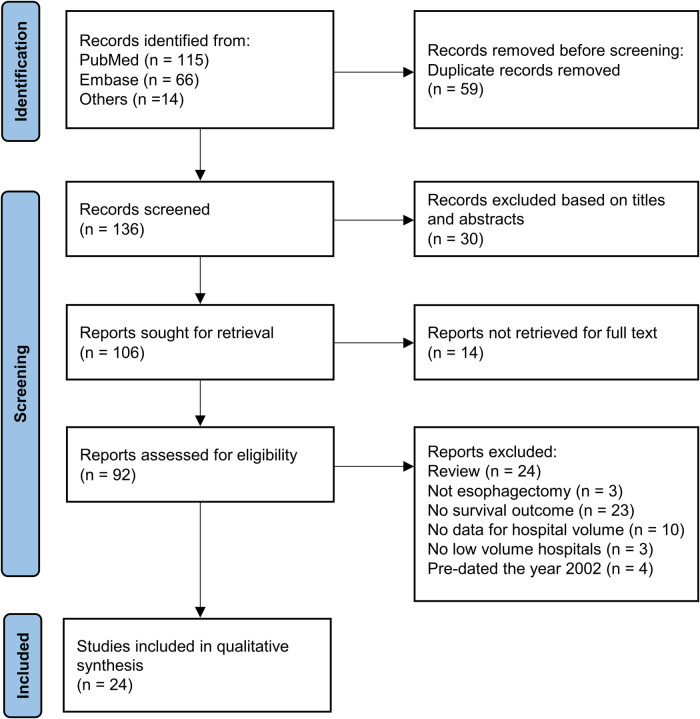
The PRIMSA flow diagram for included studies.

This systematic review was registered in https://doi.org/10.37766/inplasy2022.7.0023 (identifier: INPLASY202270023). Figure 1 shows the process of literature selection. We retrieved 115 articles from PubMed and 66 from Embase; of these, 136 studies were retained for primary selection after 59 duplicate studies were excluded. After screening of titles and abstracts, 30 studies were excluded. Among the remaining 106 articles, which were related to the volume-outcome relationship in esophageal cancer surgery, we further excluded 24 reviews without primary data, three articles not related to esophagectomy, 23 articles without data of long-term survival, 10 articles without data of hospital volume, three articles without data of low-volume hospitals, four articles published before 2002. Finally, 24 studies published from 2002 to May 2022 with 113,014 participants were included in the meta-analysis.

Among the 24 included studies, six were from the United States (6–8, 21–23), four from Sweden (9, 15, 24, 25), three each from Australia (26–28) and Netherlands (29–31), two each from Japan (32, 33) and England (14, 34), and one each from China (35), Korea (36), Brazil (37), and Canada (38) (Table 1). The longest follow-up period was 24 years.

**Table 1:** Basic characteristics of all included studies for meta-analysis on the relation between hospital volume and outcome of esophagectomies for cancer.

Author, year	Year	Country	Study	Population	Age, years	Male	Exposure	Hospital volume		Volume grouping	The longest	Survival	Hospital
[Ref]			*P*eriod			(%)		High	Low	Category	Follow-up, year	After	Number
Dikken (4)	2012	Netherlands	1989–2009	10,025	NR	76.0%	HV	≥21	≤5	Quartiles	3 years	Surgery	44
Van de Poll-Fanse (5)	2011	Netherlands	1995–2006	638	66.0	76.5%	HV	15–20	<4	Tertiles	3 years	Surgery	NR
Yang (10)	2018	USA	2004–2013	2445	62.0	90.6%	HV	3.1–15.8	0.1–1.0	Tertiles	11 years	Surgery	450
Coupland (11)	2013	England	2004–2008	5403	NR	71.9%	HV	≥80	<20	Quintiles	6 years	Surgery	NR
Derogar (13)	2013	Sweden	1987–2005	1335	66.0	74.0%	HV	≥17	≤8	Tertiles	24 years	Surgery	NR
Patel (26)	2021	USA	2006–2013	11,739	62.0–63.0	85.1%	HV	>6	≤6	Dichotomies	5 years	Surgery	1018
Han (27)	2020	USA	2004–2016	37,695	NR	NR	HV	≥25	<5	Quintiles	5 years	Surgery	NR
Gasper (28)	2009	USA	1995–2004	2404	NR	75.9%	HV	>6	<2	Quintiles	5 years	Surgery	NR
Bilimoria (29)	2008	USA	1994–1999	12,246	64.0–65.0	NR	HV	>15	<3	Quintiles	6 years	Surgery	1154
Birkmeyer (30)	2007	USA	1992–2002	822	NR	79.6%	HV	>14	<4	Tertiles	5 years	Surgery	206
Sundelof (31)	2008	Sweden	1994–1997	232	67.0	83.2%	HV	≥10	6–9	Dichotomies	10 years	Surgery	33
Rouvelas (32)	2007	Sweden	1987–2000	1199	65.0–66.0	71.9%	HV	≥10	<10	Dichotomies	17 years	Surgery	53
Wenner (33)	2005	Sweden	1987–1996	1429	66.0–67.0	72.8%	HV	>15	<5	Tertiles	13 years	Surgery	74
Narendra (34)	2021	Australia	2001–2015	1167	NR	NR	HV	≥6	NR	Dichotomies	5 years	Surgery	24
Smith (35)	2014	Australia	2001–2008	908	NR	80.5%	HV	>6	≤6	Dichotomies	9 years	Surgery	42
Stavrou (36)	2010	Australia	2000–2005	321	NR	74.0%	HV	>20	≤10	Tertiles	3 years	Surgery	NR
Verhoef (37)	2007	Netherlands	1994–2002	213	NR	69.1%	HV	≥20	<20	Dichotomies	10 years	Surgery	18
Taniyama (38)	2021	Japan	2006–2013	3578	NR	83.5%	HV	54–70	≤10	Tertiles	10 years	Surgery	96
Ioka (39)	2007	Japan	1994–1998	2961	NR	NR	HV	>43	<8	Quartiles	5 years	Surgery	143
Bachmann (40)	2002	England	1996–1997	781	NR	NR	HV	60–83	7–32	Tertiles	3 years	Surgery	23
Hsu (41)	2014	China	2008–2011	2151	55.2	94.1%	HV	>22	≤22	Dichotomies	3 years	Surgery	58
Kim (42)	2021	Korea	2004–2017	11,346	64.2	92.6%	HV	≥48	<12	Tertiles	5 years	Surgery	122
Duarte (43)	2020	Brazil	2000–2013	1347	NR	84.9%	HV	>8	<5	Dichotomies	5 years	Surgery	NR
Simunovic (44)	2006	Canada	1990–2000	629	63.0–65.0	NR	HV	≥44	≤7	Quartiles	10 years	Surgery	68

Ref, reference.

### 3.2. Quality assessment

The quality of the included studies was assessed using the Newcastle-Ottawa Scale. The median Newcastle-Ottawa Scale score of the included studies was 7, with a range of 6–9 (Table 2).

**Table 2:** Quality assessment of all included studies by Newcastle-Ottawa scale.

Study	Selection				Comparability	Outcome			Total score
	I	II	III	IV	V	VI	VII	VIII	
Dikken 2012 (29)		★	★	★	★★	★	★	★	8
Van de Poll-Fanse 2011 (30)	★	★	★		★★	★	★	★	8
Yang 2019 (21)	★	★	★		★	★	★	★	7
Coupland 2013 (14)	★	★	★	★	★★	★	★	★	9
Derogar 2013 (15)	★	★	★	★	★★	★	★	★	9
Patel 2022 (6)	★	★	★		★★	★	★	★	8
Han 2021 (7)	★	★	★	★	★★	★	★	★	9
Gasper 2009 (8)	★	★	★	★	★★	★	★	★	9
Bilimoria 2008 (22)	★	★	★	★	★★	★	★	★	9
Birkmeyer 2007 (23)	★	★	★	★	★★	★	★	★	9
Sundelof 2008 (24)			★	★	★★	★	★	★	7
Rouvelas 2007 (9)			★	★	★★	★	★		6
Wenner 2005 (25)		★	★	★	★★		★		6
Narendra 2021 (26)		★	★	★	★	★	★		6
Smith 2014 (27)		★	★	★	★★	★	★	★	8
Stavrou 2010 (28)			★	★	★★	★	★	★	7
Verhoef 2007 (31)	★	★	★	★	★★	★	★		8
Taniyama 2021 (32)	★	★		★	★★	★	★		7
Ioka 2007 (33)			★	★	★★		★	★	6
Bachmann 2002 (34)	★	★	★	★	★	★	★	★	8
Hsu 2014 (35)			★	★	★★	★	★	★	7
Kim 2021 (36)	★	★	★	★	★	★	★		7
Duarte 2020 (37)		★	★	★	★	★	★		6
Simunovic 2006 (38)		★	★	★	★	★	★		6

*One score. I, representativeness of the exposed cohorts; II, selection of the non-exposed cohorts; III, ascertainment of exposure; IV, demonstration that outcome of interest was not present at start of study of interest; V, comparability of cohorts on the basis of the design or analysis; VI, assessment of outcomes; VII, was follow-up long enough for outcomes to occur; VIII, adequacy of follow-up of cohorts.

### 3.3. Long-term os in relation to hospital volume

**Figure 2:**
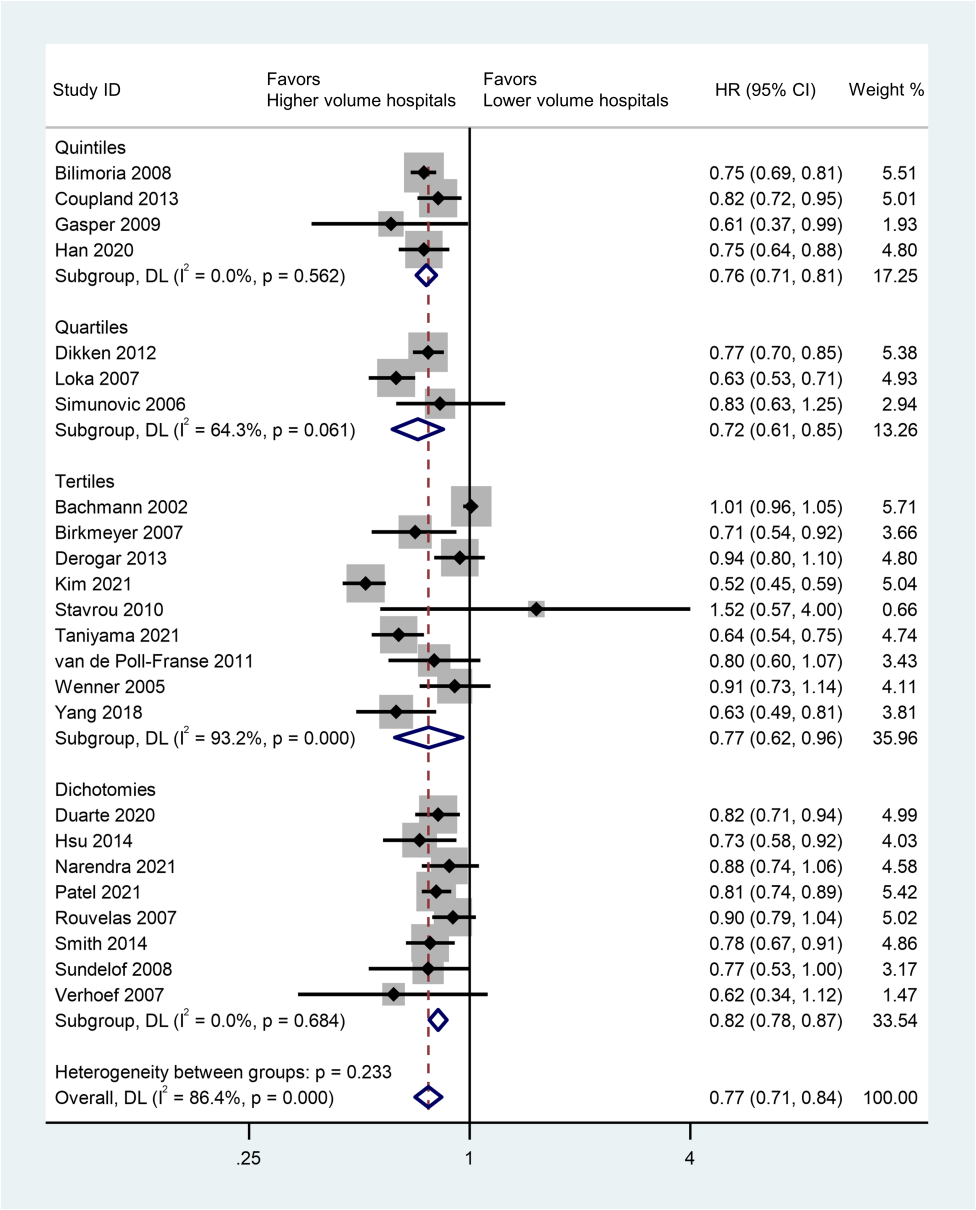
Forest plot of long-term survivals following esophagectomy comparing high- with low-volume hospitals (reference) according to volume grouping.

A total of 24 studies was included to assess the impact of high-volume vs. low-volume hospitals on long-term overall survival after esophagectomy. Regarding to the longest period of follow-ups, high-volume hospitals showed significantly better overall survival than low-volume hospitals (HR: 0.77; 95% CI: 0.71–0.84, *P* < 0.01) (Figure 2).

Next, we analyzed the pooled HRs of OS (high-volume hospital vs. low-volume hospital) for multiple cutoff values (Table 3). Consistent results were found that high-volume hospitals showed a significant improvement in OS after esophagectomy than their low-volume counterparts (all *P* ≤ 0.05).

**Table 3:** Comparisons of the overall survivals between high- and low-volume hospitals by different cutoff values of hospital volume.

Cutoff values of hospital volume (CV) HVH (≥CV) vs. LVH (<CV)	No. of studies	No. of patients	Effect estimate		
			HR	(95% CI)	*P* value
5	6	55,152	0.76	0.71–0.80	<0.001
6	11	80,408	0.79	0.75–0.84	<0.001
7	8	66,606	0.79	0.73–0.85	<0.001
8	9	67,261	0.79	0.74–0.84	<0.001
9	10	68,596	0.78	0.74–0.83	<0.001
10	12	74,347	0.77	0.72–0.83	<0.001
11	11	73,148	0.77	0.72–0.83	<0.001
12–14	12	84,494	0.75	0.68–0.83	<0.001
15	11	83,672	0.75	0.68–0.84	<0.001
16	9	80,741	0.72	0.65–0.80	<0.001
17	8	68,494	0.72	0.63–0.81	<0.001
18–19	7	67,159	0.71	0.61–0.82	<0.001
20	9	77,976	0.71	0.63–0.81	<0.001
21	8	71,427	0.72	0.63–0.82	<0.001
22	7	63,232	0.64	0.56–0.73	<0.001
23–25	6	61,081	0.70	0.60–0.82	<0.001
26–32	5	23,386	0.69	0.57–0.84	<0.001
33–43	6	24,167	0.75	0.59–0.95	0.02
44	5	21,737	0.74	0.55–1.00	0.05

CI, confidence interval; HR, hazard ratio; HVH, high-volume hospital; LVH, low-volume hospital; No., number.

### 3.4. Subgroup analysis

Subgroup analysis was conducted based on volume grouping category in Figure 2. A significant improvement in OS after esophagectomy was observed in high-volume hospitals as compared to that in their low-volume counterparts in each volume grouping category. The pooled HRs were 0.76 (95% CI: 0.71–0.81) for quintiles, 0.72 (95% CI: 0.61–0.85) for quartiles, 0.77 (95% CI:0.62–0.96) for tertiles, and 0.82 (95% CI:0.78–0.87) for dichotomies, respectively (Figure 2, Table 4).

**Table 4:** Subgroup analyses of comparisons of the overall survivals between high- and low-volume hospitals.

Subgroup HVH vs. LVH	No. of studies	No. of patients	Effect estimate	
			HR (95% CI)	*P* value
Total	24	113,014	0.77 (0.71–0.84)	<0.001
**Volume group**				
Dichotomies	8	18,956	0.82 (0.78–0.87)	<0.001
Tertiles	9	22,695	0.77 (0.62–0.96)	0.02
Quartiles	3	13,615	0.72 (0.61–0.85)	<0.001
Quintiles	4	57,748	0.76 (0.71–0.81)	<0.001
**Sample size**				
>5,000	6	88,454	0.73 (0.65–0.82)	<0.001
<5,000	18	24,560	0.79 (0.72–0.87)	<0.001
**Study country**				
Western countries	20	98,381	0.82 (0.76–0.88)	<0.001
Eastern countries	4	20,036	0.61 (0.53–0.70)	<0.001
**Year of publication**				
2002–2012	13	33,900	0.80 (0.70–0.90)	<0.001
2013–2022	11	79,114	0.75 (0.67–0.83)	<0.001
**Follow-up**				
Longest follow-up ≥10 years	8	11,060	0.79 (0.69–0.91)	<0.001
Longest follow-up <10 years	16	101,954	0.76 (0.69–0.85)	<0.001
**Study quality**				
High	19	107,243	0.74 (0.67–0.83)	<0.001
Moderate	5	5771	0.87 (0.80–0.94)	<0.001

CI, confidence interval; HR, hazard ratio; HVH, high-volume hospital; LVH, low-volume hospital; No., number.

In addition, we carried out subgroup analyses in relation to sample size, study country, year of publication, follow-up or study quality. Overall, the results were robust and that patients with esophagectomy significantly benefited from high-volume hospitals than from low-volume hospitals (Table 3).

### 3.5. Sensitivity analyses

Sensitivity analyses with the leave-one-out method further revealed the consistent results, which were observed a significant improvement in OS after esophagectomy in high-volume hospitals as compared to that in their low-volume counterparts, with HRs ranging from 0.75 (95% CI: 0.68–0.83) to 0.79 (95% CI: 0.73–0.85) (Table 5).

**Table 5:** Sensitivity analysis using leave-one-out method for overall survival of high-volume hospitals vs. low-volume hospitals.

Given named study is omitted	Hazard ratio	95% CI	*P* value
Dikken (29)	0.77	0.70–0.84	<0.001
Van de Poll-Fanse (30)	0.77	0.71–0.84	<0.001
Yang (21)	0.78	0.71–0.85	<0.001
Coupland (14)	0.77	0.70–0.84	<0.001
Derogar (15)	0.76	0.70–0.83	<0.001
Patel (6)	0.77	0.70–0.84	<0.001
Han (7)	0.77	0.71–0.84	<0.001
Gasper (8)	0.75	0.68–0.83	<0.001
Bilimoria (22)	0.77	0.70–0.85	<0.001
Birkmeyer (23)	0.77	0.71–0.84	<0.001
Sundelof (24)	0.77	0.70–0.84	<0.001
Rouvelas (9)	0.76	0.70–0.84	<0.001
Wenner (25)	0.77	0.70–0.84	<0.001
Narendra (26)	0.77	0.70–0.84	<0.001
Smith (27)	0.77	0.70–0.84	<0.001
Stavrou (28)	0.77	0.70–0.84	<0.001
Verhoef (31)	0.77	0.71–0.84	<0.001
Taniyama (32)	0.78	0.71–0.85	0.02
Ioka (33)	0.78	0.72–0.85	0.05
Bachmann (34)	0.76	0.71–0.81	<0.001
Hsu (35)	0.77	0.71–0.84	<0.001
Kim (36)	0.79	0.73–0.85	<0.001
Duarte (37)	0.77	0.70–0.84	<0.001
Simunovic (38)	0.77	0.70–0.84	<0.001

CI, confidence interval.

### 3.6. Publication bias

**Figure 3:**
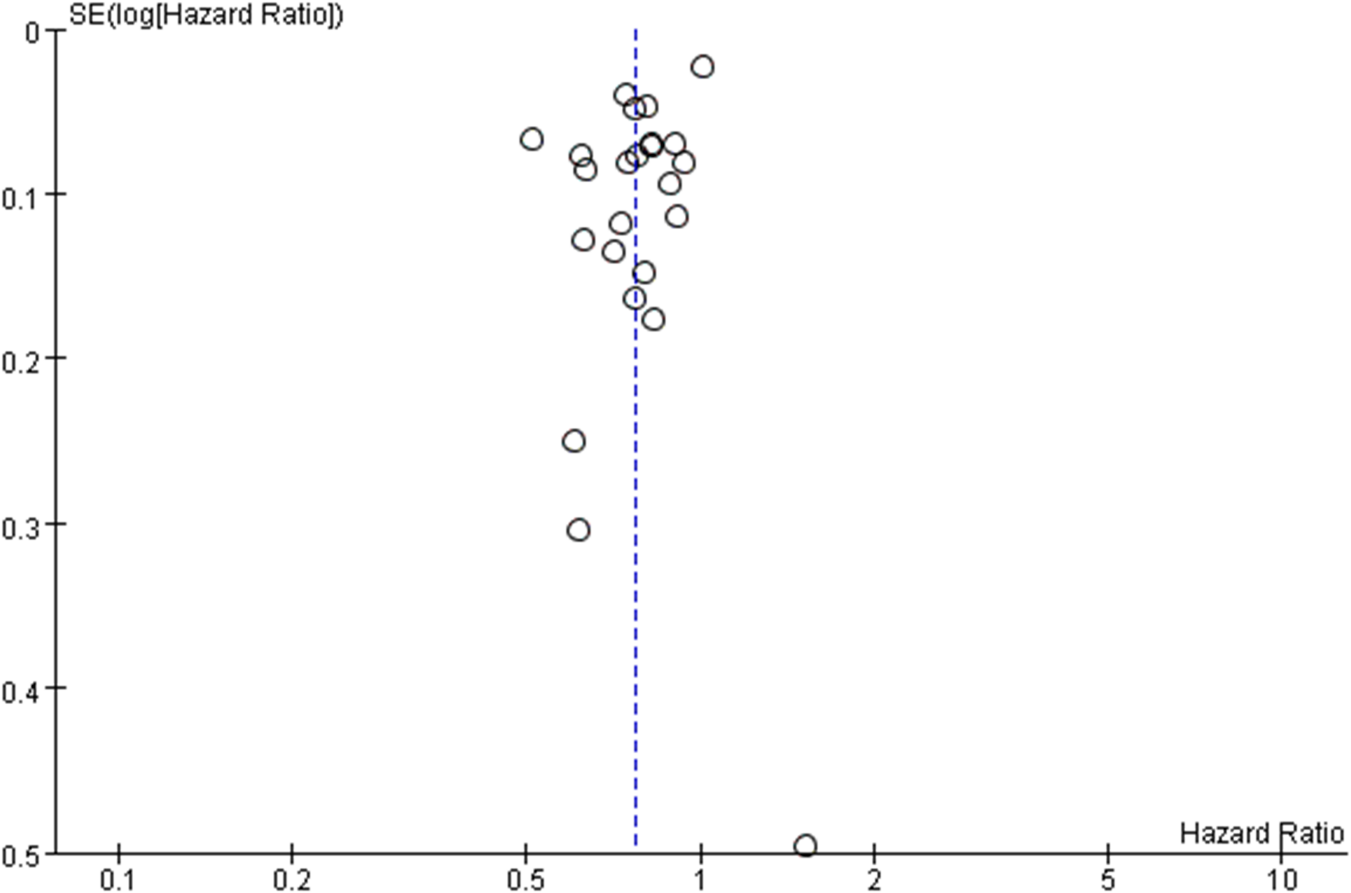
Funnel plot of survival benefit following esophagectomy comparing high- with low-volume hospital (reference).

We further assessed the publication bias (Figure 3). Because of the relatively small number of included studies in some volume grouping category meta-analyses, we consider that publication bias should exist.

## 4. Discussion

This meta-analysis outlined the most up-to-date evidence on the relationship between hospital volume and long-term survival outcomes in esophagectomy. We found for the first time that centralization of esophagectomy in high-volume hospitals improved OS as compared to that in low-volume hospitals and patients with esophageal cancer will benefit from an esophagectomy conducted in a higher volume hospital than in a lower one, whether in total or in volume grouping category. However, we were still unable to decide the optimal cutoff value of dividing high- and low-volume hospitals in current study.

Centralization of esophageal cancer surgery has been common in the Netherlands, England, and Canada (18, 39, 40), Comparing a centralized country (England) with a non-centralized country (U.S.), a previous study of 13,291 patients illustrated a lower in-hospital mortality in England hospitals than those in the U.S. (4.2% vs. 5.5%) (41). Regarding this, centralization is urgently required, in terms of high-volume hospitals with sufficient surgical volumes, skillful interdisciplinary teams, to provide the optimal treatment for patients with esophageal cancer.

Although the reasons why high-volume hospitals are associated with better long-term survival are still not fully understood, high-volume hospitals may provide patients with better multidisciplinary teams, more comprehensive preoperative examinations, more accurate preoperative diagnosis, perioperative management, and high-quality surgical care, more specialized surgeons who have more consistent skills of performing curable operations for esophageal cancer patients (42–45). Compared with low-volume hospitals, high-volume hospitals not only have a lower complication rate after esophagectomies, but also the ability of managing complications (46). In addition, the applications of neoadjuvant chemoradiation, perioperative chemotherapy, and postoperatively follow-up can improve long-term outcomes after esophagectomies; therefore, high-volume hospitals are more likely to provide a better overall cancer therapy and care, and the size of hospital volume may serve as a significant indicator of the overall medical quality and health care (47).

Unfortunately, it is difficult for patients to know the overall quality of nearby hospitals. Based on the main findings of current study, patients can select relatively higher volume hospitals nearby. Considering the importance of such knowledges, policy makers should make efforts to educate people for selecting the optimal hospitals for the treatments of specific diseases (e.g., esophagectomy for esophageal cancer), through public reporting systems.

Our study still has limitations. First, this study has the potential for selection bias of individual studies because of the original data, even with case mix adjustment. Second, all the included studies were observational and retrospective. Third, some of the included studies used the same database (e.g., Sweden), and some participants might be overlapped, even though the study period were different; however, sensitivity analyses of a leave-one-out method confirmed that all the current results were robust. Fourth, as some of the data in the included studies were obtained from the National Cancer Registry, some details of the surgery, such as surgical approach and the extent of lymph nodes dissection, were unknown. Fifth, the volume grouping categories of the annual hospital volumes across the included studies varied greatly, and there was still no optimal threshold, and the main findings of current study thus need to be verified in further studies.

## 5. Conclusion

In summary, high-volume hospitals significantly improved long-term OS of patients with esophageal cancer after esophagectomy as compared to their low-volume counterparts. Esophagectomy should be centralized in high-volume hospitals.

## Data Availability

The original contributions presented in the study are included in the article, further inquiries can be directed to the corresponding author.

## References

[1] Sheetz KH Dimick JB Nathan H. Centralization of high-risk cancer surgery within existing hospital systems. J Clin Oncol. (2019) 37(34):3234–42. 10.1200/JCO.18.02035 31251691 PMC7351344

[2] Balzano G Guarneri G Pecorelli N Paiella S Rancoita PMV Bassi C Modelling centralization of pancreatic surgery in a nationwide analysis. Br J Surg. (2020) 107(11):1510–9. 10.1002/bjs.11716 32592514

[3] van Putten M Nelen SD Lemmens VEPP Stoot JHMB Hartgrink HH Gisbertz SS Overall survival before and after centralization of gastric cancer surgery in The Netherlands. Br J Surg. (2018) 105(13):1807–15. 10.1002/bjs.10931 30132789

[4] Asplund J Mattsson F Plecka-Ostlund M Markar SR Lagergren J. Annual surgeon and hospital volume of gastrectomy and gastric adenocarcinoma survival in a population-based cohort study. Acta Oncol. (2022) 61(4):425–32. 10.1080/0284186X.2022.2025612 35023804

[5] Lagergren J Lagergren P. Recent developments in esophageal adenocarcinoma. CA Cancer J Clin. (2013) 63(4):232–48. 10.3322/caac.21185 23818335

[6] Patel DC Jeffrey Yang CF He H Liou DZ Backhus LM Lui NS Influence of facility volume on long-term survival of patients undergoing esophagectomy for esophageal cancer. J Thorac Cardiovasc Surg. (2022) 163(4):1536–1546 e1533. 10.1016/j.jtcvs.2021.05.048 34247867

[7] Han S Kolb JM Hosokawa P Friedman C Fox C Scott FI The volume-outcome effect calls for centralization of care in esophageal adenocarcinoma: results from a large national cancer registry. Am J Gastroenterol. (2021) 116(4):811–5. 10.14309/ajg.0000000000001046 33982952

[8] Gasper WJ Glidden DV Jin C Way LW Patti MG. Has recognition of the relationship between mortality rates and hospital volume for major cancer surgery in California made a difference?: a follow-up analysis of another decade. Ann Surg. (2009) 250(3):472–83. 10.1097/SLA.0b013e3181b47c79 19730178

[9] Rouvelas I Lindblad M Zeng W Viklund P Ye W Lagergren J. Impact of hospital volume on long-term survival after esophageal cancer surgery. Arch Surg. (2007) 142(2):113–7; discussion 118. 10.1001/archsurg.142.2.113 17309961

[10] Yang H Liu H Chen Y Zhu C Fang W Yu Z Long-term efficacy of neoadjuvant chemoradiotherapy plus surgery for the treatment of locally advanced esophageal squamous cell carcinoma: the NEOCRTEC5010 randomized clinical trial. JAMA Surg. (2021) 156(8):721–9. 10.1001/jamasurg.2021.2373 34160577 PMC8223138

[11] Yu S Zhang W Ni W Xiao Z Wang Q Zhou Z A propensity-score matching analysis comparing long-term survival of surgery alone and postoperative treatment for patients in node positive or stage III esophageal squamous cell carcinoma after R0 esophagectomy. Radiother Oncol. (2019) 140:159–66. 10.1016/j.radonc.2019.06.020 31302346

[12] He S Xu J Liu X Zhen Y. Advances and challenges in the treatment of esophageal cancer. Acta Pharm Sin B. (2021) 11(11):3379–92. 10.1016/j.apsb.2021.03.008 34900524 PMC8642427

[13] Lagergren J Smyth E Cunningham D Lagergren P. Oesophageal cancer. Lancet. (2017) 390(10110):2383–96. 10.1016/S0140-6736(17)31462-9 28648400

[14] Coupland VH Lagergren J Luchtenborg M Jack RH Allum W Holmberg L Hospital volume, proportion resected and mortality from oesophageal and gastric cancer: a population-based study in England, 2004-2008. Gut. (2013) 62(7):961–6. 10.1136/gutjnl-2012-303008 23086798

[15] Derogar M Sadr-Azodi O Johar A Lagergren P Lagergren J. Hospital and surgeon volume in relation to survival after esophageal cancer surgery in a population-based study. J Clin Oncol. (2013) 31(5):551–7. 10.1200/JCO.2012.46.1517 23295792

[16] Gillison E. W. Powell J. McConkey C. C. Spychal R. T. Surgical workload and outcome after resection for carcinoma of the oesophagus and cardia. Br J Surg. (2002) 89(3):344–8. 10.1046/j.0007-1323.2001.02015.x 11872061

[17] Wang Q Nasu M Fukunaga T Nojiri S Zhang C-D Mine S. Association of hospital volume and long-term survival after esophagectomy: a systematic review and meta-analysis. INPLASY. (2022). 10.37766/inplasy2022.7.0023 PMC10160622 37151870

[18] Page MJ McKenzie JE Bossuyt PM Boutron I Hoffmann TC Mulrow CD The PRISMA 2020 statement: an updated guideline for reporting systematic reviews. Br Med J. (2021) 372:n71. 10.1136/bmj.n71 33782057 PMC8005924

[19] Stang A. Critical evaluation of the Newcastle-Ottawa scale for the assessment of the quality of nonrandomized studies in meta-analyses. Eur J Epidemiol. (2010) 25(9):603–5. 10.1007/s10654-010-9491-z 20652370

[20] Higgins J Thompson S Deeks J Altman D. Measuring inconsistency in meta-analyses. Br Med J. (2003) 327(7414):557–60. 10.1136/bmj.327.7414.557 12958120 PMC192859

[21] Yang GQ Mhaskar R Rishi A Naghavi AO Frakes JM Almhanna K Intensity-modulated radiotherapy at high-volume centers improves survival in patients with esophageal adenocarcinoma receiving trimodality therapy. Dis Esophagus. (2019) 32(8):doy124. 10.1093/dote/doy124 30597022

[22] Bilimoria KY Bentrem DJ Feinglass JM Stewart AK Winchester DP Talamonti MS Directing surgical quality improvement initiatives: comparison of perioperative mortality and long-term survival for cancer surgery. J Clin Oncol. (2008) 26(28):4626–33. 10.1200/JCO.2007.15.6356 18574159

[23] Birkmeyer JD Sun Y Wong SL Stukel TA. Hospital volume and late survival after cancer surgery. Ann Surg. (2007) 245(5):777–83. 10.1097/01.sla.0000252402.33814.dd 17457171 PMC1877074

[24] Sundelof M Lagergren J Ye W. Surgical factors influencing outcomes in patients resected for cancer of the esophagus or gastric cardia. World J Surg. (2008) 32(11):2357–65. 10.1007/s00268-008-9698-2 18716831

[25] Wenner J Zilling T Bladström A Alvegård T. The influence of surgical volume on hospital mortality and 5-year survival for carcinoma of the oesophagus and gastric cardia. Anticancer Res. (2005) 25(1B):419–24. 15816605

[26] Narendra A Baade PD Aitken JF Fawcett J Leggett B Leggett C Hospital characteristics associated with better “quality of surgery” and survival following oesophagogastric cancer surgery in Queensland: a population-level study. ANZ J Surg. (2021) 91(3):323–8. 10.1111/ans.16397 33155394

[27] Smith RC Creighton N Lord RV Merrett ND Keogh GW Liauw WS Survival, mortality and morbidity outcomes after oesophagogastric cancer surgery in New South Wales, 2001-2008. Med J Aust. (2014) 200(7):408–13. 10.5694/mja13.11182 24794674

[28] Stavrou EP Smith GS Baker DF. Surgical outcomes associated with oesophagectomy in New South Wales: an investigation of hospital volume. J Gastrointest Surg. (2010) 14(6):951–7. 10.1007/s11605-010-1198-7 20414814

[29] Dikken JL Dassen AE Lemmens VE Putter H Krijnen P van der Geest L Effect of hospital volume on postoperative mortality and survival after oesophageal and gastric cancer surgery in The Netherlands between 1989 and 2009. Eur J Cancer. (2012) 48(7):1004–13. 10.1016/j.ejca.2012.02.064 22456179

[30] van de Poll-Franse LV Lemmens VE Roukema JA Coebergh JW Nieuwenhuijzen GA. Impact of concentration of oesophageal and gastric cardia cancer surgery on long-term population-based survival. Br J Surg. (2011) 98(7):956–63. 10.1002/bjs.7493 21509748

[31] Verhoef C van de Weyer R Schaapveld M Bastiaannet E Plukker JT. Better survival in patients with esophageal cancer after surgical treatment in university hospitals: a plea for performance by surgical oncologists. Ann Surg Oncol. (2007) 14(5):1678–87. 10.1245/s10434-006-9333-0 17294070 PMC1914254

[32] Taniyama Y Tabuchi T Ohno Y Morishima T Okawa S Koyama S Hospital surgical volume and 3-year mortality in severe prognosis cancers: a population-based study using cancer registry data. J Epidemiol. (2021) 31(1):52–8. 10.2188/jea.JE20190242 31932528 PMC7738649

[33] Ioka A Tsukuma H Ajiki W Oshima A. Hospital procedure volume and survival of cancer patients in Osaka, Japan: a population-based study with latest cases. Jpn J Clin Oncol. (2007) 37(7):544–53. 10.1093/jjco/hym052 17720740

[34] Bachmann M Alderson D Edwards D Wotton S Bedford C Peters T Cohort study in south and west England of the influence of specialization on the management and outcome of patients with oesophageal and gastric cancers. Br J Surg. (2002) 89(7):914–22. 10.1046/j.1365-2168.2002.02135.x 12081743

[35] Hsu PK Chen HS Wu SC Wang BY Liu CY Shih CH Impact of hospital volume on long-term survival after resection for oesophageal cancer: a population-based study in taiwandagger. Eur J Cardiothorac Surg. (2014) 46(6):e127–135; discussion e135. 10.1093/ejcts/ezu377 25281656

[36] Kim BR Jang EJ Jo J Lee H Jang DY Ryu HG. The association between hospital case-volume and postoperative outcomes after esophageal cancer surgery: a population-based retrospective cohort study. Thorac Cancer. (2021) 12(18):2487–93. 10.1111/1759-7714.14096 34355527 PMC8447910

[37] Duarte MBO Pereira EB Lopes LR Andreollo NA Carvalheira JBC. Chemoradiotherapy with or without surgery for esophageal squamous cancer according to hospital volume. JCO Glob Oncol. (2020) 6:828–36. 10.1200/JGO.19.00360 32552112 PMC7328122

[38] Simunovic M Rempel E Thériault M Coates A Whelan T Holowaty E Influence of hospital characteristics on operative death and survival of patients after major cancer surgery in Ontario. Can J Surg. (2006) 49(4):251–8. 16948883 PMC3207572

[39] Varghese TK Jr. Wood DE Farjah F Oelschlager BK Symons RG MacLeod KE Variation in esophagectomy outcomes in hospitals meeting leapfrog volume outcome standards. Ann Thorac Surg. (2011) 91(4):1003–9; discussion 1009–1010. 10.1016/j.athoracsur.2010.11.006 21440116

[40] Chang AC. Centralizing esophagectomy to improve outcomes and enhance clinical research: invited expert review. Ann Thorac Surg. (2018) 106(3):916–23. 10.1016/j.athoracsur.2018.04.004 29738757

[41] Munasinghe A Markar SR Mamidanna R Darzi AW Faiz OD Hanna GB Is it time to centralize high-risk cancer care in the United States? Comparison of outcomes of esophagectomy between England and the United States. Ann Surg. (2015) 262(1):79–85. 10.1097/SLA.0000000000000805 24979602

[42] Van den Broeck T Oprea-Lager D Moris L Kailavasan M Briers E Cornford P A systematic review of the impact of surgeon and hospital caseload volume on oncological and nononcological outcomes after radical prostatectomy for nonmetastatic prostate cancer. Eur Urol. (2021) 80(5):531–45. 10.1016/j.eururo.2021.04.028 33962808

[43] Khanna A Saarela O Lawson K Finelli A Haber GP Lee B Hospital quality metrics for radical cystectomy: disease specific and correlated to mortality outcomes. J Urol. (2019) 202(3):490–7. 10.1097/JU.0000000000000282 31009290

[44] Wright JD Chen L Hou JY Burke WM Tergas AI Ananth CV Association of hospital volume and quality of care with survival for ovarian cancer. Obstet Gynecol. (2017) 130(3):545–53. 10.1097/AOG.0000000000002164 28796677 PMC5650072

[45] Ghaferi AA Birkmeyer JD Dimick JB. Complications, failure to rescue, and mortality with major inpatient surgery in medicare patients. Ann Surg. (2009) 250(6):1029–34. 10.1097/sla.0b013e3181bef697 19953723

[46] Ghaferi A Birkmeyer J Dimick J. Hospital volume and failure to rescue with high-risk surgery. Med Care. (2011) 49(12):1076–81. 10.1097/MLR.0b013e3182329b97 22002649

[47] Eskander A Irish J Groome PA Freeman J Gullane P Gilbert R Volume-outcome relationships for head and neck cancer surgery in a universal health care system. Laryngoscope. (2014) 124(9):2081–8. 10.1002/lary.24704 24706437

